# Diel Cycle Proteomics: Illuminating Molecular Dynamics in Purple Bacteria for Optimized Biotechnological Applications

**DOI:** 10.3390/ijms25052934

**Published:** 2024-03-02

**Authors:** Sabine Matallana-Surget, Augustin Geron, Corentin Decroo, Ruddy Wattiez

**Affiliations:** 1 Division of Biological and Environmental Sciences, Faculty of Natural Sciences, University of Stirling, Stirling FK9 4LA, UK; 2 Proteomic and Microbiology Department, University of Mons, B-7000 Mons, Belgium

**Keywords:** circadian clock, diel cycle, kai operon, purple bacteria, proteomics

## Abstract

Circadian rhythms, characterized by approximately 24 h cycles, play a pivotal role in enabling various organisms to synchronize their biological activities with daily variations. While ubiquitous in Eukaryotes, circadian clocks remain exclusively characterized in *Cyanobacteria* among Prokaryotes. These rhythms are regulated by a core oscillator, which is controlled by a cluster of three genes: *kaiA*, *kaiB*, and *kaiC*. Interestingly, recent studies revealed rhythmic activities, potentially tied to a circadian clock, in other Prokaryotes, including purple bacteria such as *Rhodospirillum rubrum*, known for its applications in fuel and plastic bioproduction. However, the pivotal question of how light and dark cycles influence protein dynamics and the expression of putative circadian clock genes remains unexplored in purple non-sulfur bacteria. Unraveling the regulation of these molecular clocks holds the key to unlocking optimal conditions for harnessing the biotechnological potential of *R. rubrum*. Understanding how its proteome responds to different light regimes—whether under continuous light or alternating light and dark cycles—could pave the way for precisely fine-tuning bioproduction processes. Here, we report for the first time the expressed proteome of *R. rubrum* grown under continuous light versus light and dark cycle conditions using a shotgun proteomic analysis. In addition, we measured the impact of light regimes on the expression of four putative circadian clock genes (*kaiB1*, *kaiB2*, *kaiC1*, *kaiC2*) at the transcriptional and translational levels using RT-qPCR and targeted proteomic (MRM-MS), respectively. The data revealed significant effects of light conditions on the overall differential regulation of the proteome, particularly during the early growth stages. Notably, several proteins were found to be differentially regulated during the light or dark period, thus impacting crucial biological processes such as energy conversion pathways and the general stress response. Furthermore, our study unveiled distinct regulation of the four *kai* genes at both the mRNA and protein levels in response to varying light conditions. Deciphering the impact of the diel cycle on purple bacteria not only enhances our understanding of their ecology but also holds promise for optimizing their applications in biotechnology, providing valuable insights into the origin and evolution of prokaryotic clock mechanisms.

## 1. Introduction

Circadian clocks are endogenous biological mechanisms that synchronize a wide array of biological processes oscillating with the diel cycle [1]. While ubiquitous in Eukaryotes, circadian clocks were initially characterized solely in oxygenic photosynthetic bacteria, specifically *Cyanobacteria*, among Prokaryotes [2,3]. The circadian clock of *Synechococcus elongatus*, a model organism in the study of cyanobacterial circadian rhythms, relies on Kai proteins (i.e., KaiA, KaiB, and KaiC), forming a core oscillator that synchronizes with environmental signals and controls gene expression [4,5]. For a more in-depth understanding of the KaiABC oscillator’s functioning and its regulatory network, please refer to reviews Cohen & Golden [6]; Swan and colleagues [7]; Snijder & Axmann [8]. The composition and functioning of Kai systems in *Cyanobacteria* exhibit significant variation, albeit being less extensively documented [9]. For example, the globally distributed marine *Prochlorococcus marinus* features a simplified clock mechanism lacking KaiA [10]. In contrast, *Synechocystis* sp. PCC 6803 encodes multiple Kai proteins, suggested to play a role in fine-tuning the core oscillator [11,12,13,14]. Importantly, the presence of Kai proteins is not exclusive to *Cyanobacteria*. Bioinformatic analyses have revealed that homologs of Kai proteins are widespread in Prokaryotes and present in purple bacteria [9,15,16]. A recent comprehensive review emphasizes the importance of circadian clocks in Prokaryotes and their potential applications in various fields, including medical research, environmental sciences, and biotechnology [17]. 

Evidence for circadian clocks in non-photosynthetic Prokaryotes has been limited, despite the overwhelming evidence of the ubiquity of cyclic mechanisms in different ecosystems. Recent investigations have unveiled circadian rhythms in *Bacillus subtilis*, a non-photosynthetic bacterium, exhibiting a synchronized 24 h light or temperature cycles with phase-specific characteristics of entrainment [18,19]. Interestingly, this bacterial species does not harbor any Kai protein homologs. Similarly, the gastrointestinal bacterium *Klebsiella aerogenes* displayed an endogenously generated, temperature-compensated circadian rhythm in swarming motility [20].

Rhythmic activities in purple non-sulfur bacteria harboring Kai homologs have been reported in three species: *Rhodopseudomonas palustris* [21], *Rhodobacter sphaeroides* [22], and *Rhodospirillum rubrum* [23]. Interestingly, purple non-sulfur bacteria also exhibit high metabolic versatility and find applications in various biotechnological fields, including bioproduction, biofertilization, and wastewater treatment [24]. 

In this study, we investigated the diel cycle response of the purple non-sulfur and nitrogen fixating *R. rubrum*, which exhibits a highly versatile metabolism capable of both photoautotrophic and heterotrophic growth [25]. This makes this bacterium particularly interesting for biotechnological applications such as bioplastic and hydrogen fuel production [26,27]. *R. rubrum* possesses two homologs of the circadian clock *kaiB* and *kaiC* genes and one potential homolog of one of the gene *kaiA* of *Synechocystis* sp. PCC 6803 [14]. Interestingly, rhythmic activity of the uptake hydrogenase (Hup), an enzyme involved in the consumption of H2, was reported using an enzyme assay [23]. The authors suggested that the rhythmic hydrogenase activity could be involved in a mechanism coordinating the energy metabolism in *R. rubrum*. However, information is still missing about the general response and the expression of *kai* genes of *R. rubrum* under the diel cycle.

**Figure 1:**
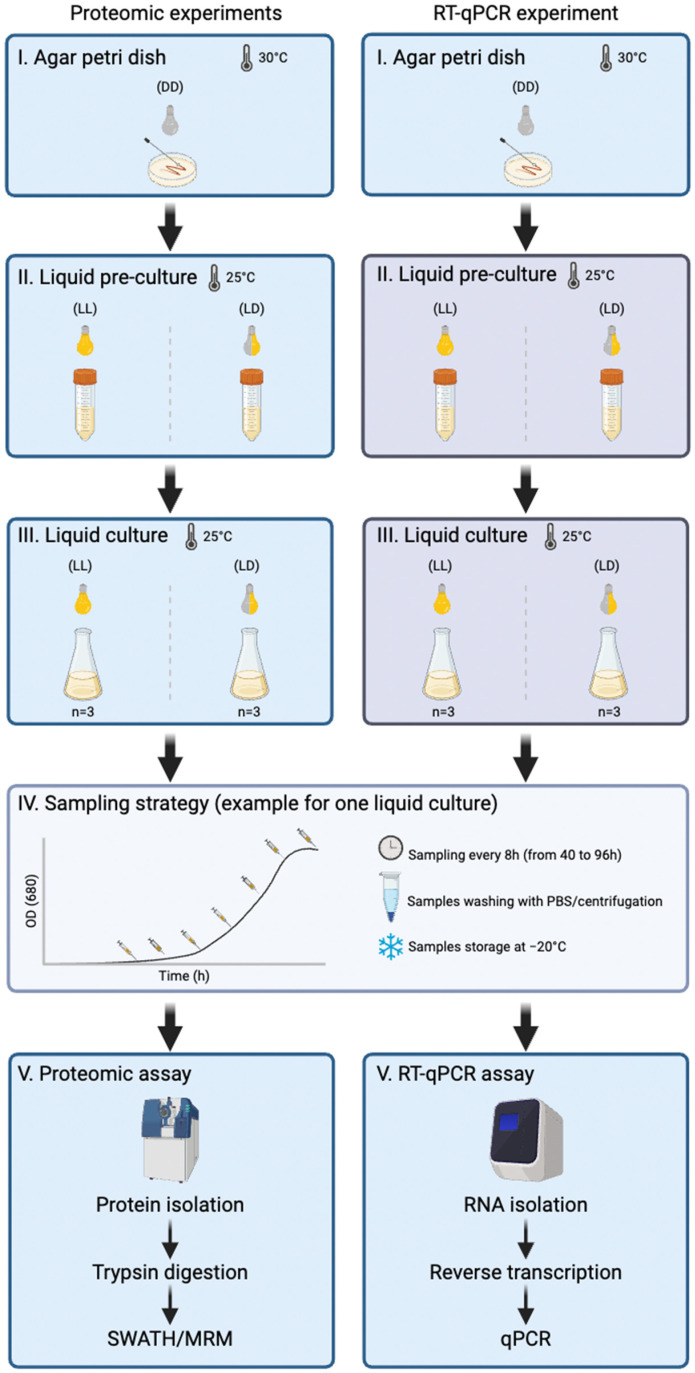
Experimental workflow for studying the molecular diel cycle in *R. rubrum*.

We conducted the first comprehensive molecular investigation to unveil the effects of light regimes on both the proteome and *kai* gene expression in *R. rubrum*. We cultivated *R. rubrum* under controlled light conditions using a phytotron, and subjected the bacterial cultures to two distinct light exposures: continuous light exposure (LL) and a 12 h light–dark cycle (LD). Using cutting-edge methodologies, we applied Shotgun Proteomics with Sequential Window Acquisition of All Theoretical Mass Spectra (SWATH-MS) analysis to precisely capture dynamic fluctuations across the entire proteome. Furthermore, we specifically examined the regulatory expression of the two *kaiB* and *kaiC* gene homologs at both the transcriptional and translational levels, using reverse transcription quantitative polymerase chain reaction (RT-qPCR) and multiple reaction monitoring (MRM-MS) (Figure 1). This comprehensive molecular study, with implications for biotechnological applications, not only enhances the understanding of *R. rubrum*’s molecular responses but more broadly provides valuable insights into the molecular clock in Prokaryotes, especially non-photosynthetic organisms.

## 2. Results and Discussion

### 2.1. Impact of Light Conditions on R. rubrum Growth 

**Figure 2:**
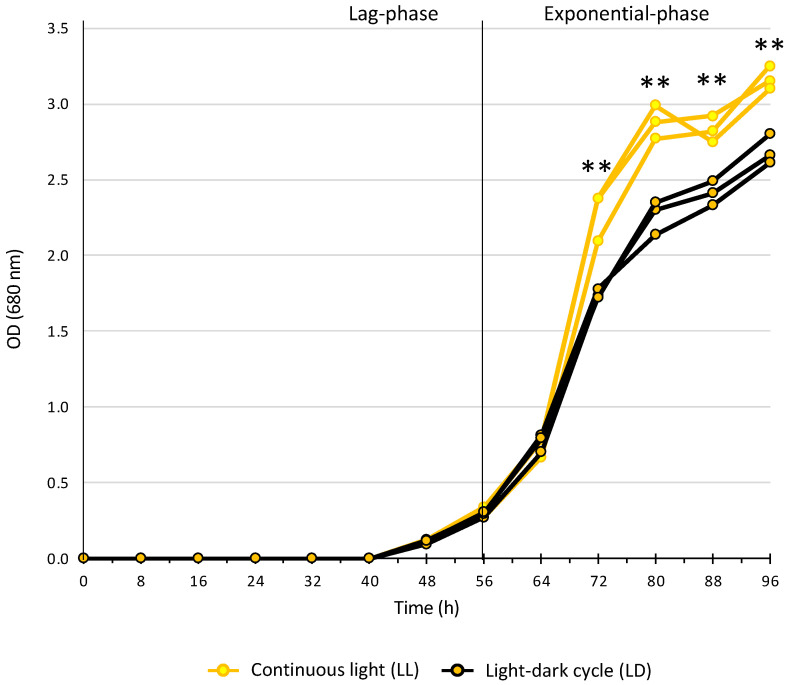
Growth curves of *R. rubrum* under different light conditions. Growth curves were computed based on optical density (OD, 680 nm). Light and dark phases in the LD condition are represented on the time axis in yellow and grey, respectively. Significant differences between LL and LD samples are shown with a ** (*p*-value ≤ 0.05) and were calculated with a *t*-test.

The impact of the light and dark cycle on bacterial growth was negligible in the lag phase and the early exponential phase (Figure 2). From 72 h, the optical density was significantly higher under continuous light exposure and reached a final average maximum of 3.16 ± 0.07 (LL) and 2.69 ± 0.09 (LD) (*p*-value = 0.002) (Figure 2). *R. rubrum* is a facultative anaerobe capable of aerobic heterotrophic growth and anaerobic photosynthesis growth [25]. In this experimental setup, heterotrophic growth was clearly favored as *R. rubrum* was in the oxic condition. However, the color of the cultures in both LL and LD conditions turned from whitish to reddish overtime, which suggests that photosynthesis was progressively activated [28].

### 2.2. Proteomic Analysis of R. rubrum under the LD Cycle

**Figure 3:**
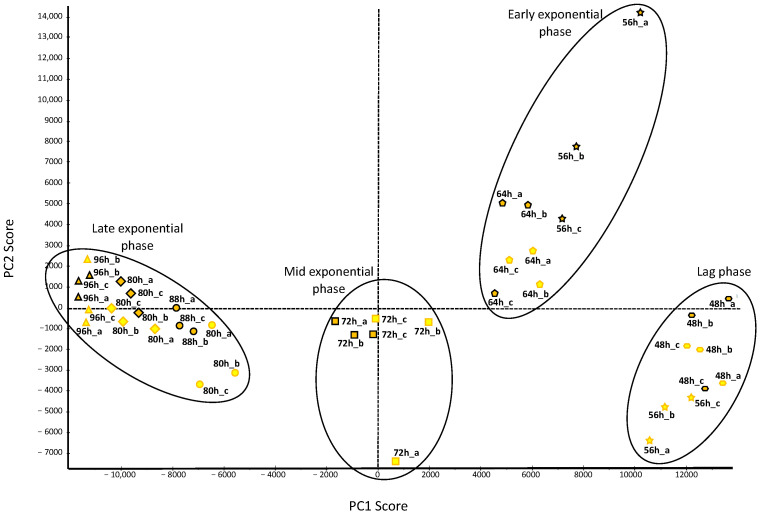
Non-supervised Pareto-PCA analysis of *R. rubrum* proteomes under different light conditions. Scatter plot of the first two components PC1 (48.7%) and PC2 (8.0%). LD proteomes are represented by orange-filled shapes with black outlines, while LL proteomes are represented by yellow-filled shapes with orange outlines. Each shape represents a sampling time point (h: hours), and ‘a’, ‘b’, and ‘c’ represent replicates.

For the first time, we present a comprehensive quantitative analysis of the entire proteome during a 56 h growth period of *R. rubrum* under LL and LD conditions. A total of 1901 unique proteins were identified, covering 50% of the predicted *R. rubrum* proteome (3835 proteins). Non-supervised Pareto-PCA analysis revealed that the LL and LD proteomes were mainly grouped based on the growth stages: (i) the lag phase proteomes (LL: 48 h and 56 h, LD: 48 h); (ii) the early exponential phase proteomes (LL: 64 h, LD: 56 h and 64 h); (iii) the mid exponential phase proteomes (LL and LD: 72 h); and (iv) the late exponential phase proteomes (LL and LD: 80 h, 88 h, and 96 h) (Figure 3). In the earliest stages (groups 1 and 2), an exception was noted where the LL and LD 56 h proteomes exhibited distinct grouping. This observation suggests a differential proteome regulation between LL and LD cultures during the early exponential phase (Figure 3). 

#### 2.2.1. Cyclic Protein Regulation

**Figure 4:**
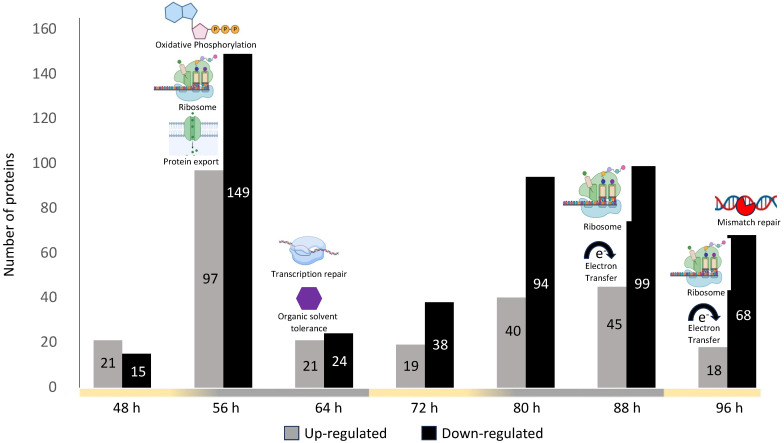
Up- and downregulated proteins in the LL condition compared to the LD condition in *R. rubrum*. Network STRING analysis results are illustrated for each subset of up- and downregulated proteins. The absence of illustration indicates that no significant functional enrichment was identified. Light and dark phases in the LD condition are represented on the time axis in yellow and grey, respectively.

We investigated the up- and downregulated proteins in LL compared to LD conditions for each sampling point. In total, 548 proteins were found to be significantly up- and/or downregulated at one or more of the seven time points (Figure 4). The time point with the highest number of regulated proteins was 56 h, featuring 97 upregulated and 149 downregulated proteins (Figure 4). This observation aligned with the PCA analysis, indicating significant separation between LL 56 h and LD 56 h proteomes (Figure 3) LL downregulated proteins were prevalent during the late exponential phase (80 h–96 h), suggesting upregulation of these functions under LD conditions (Figure 4). To enhance the interpretation of protein regulation, we categorized them into protein regulation profiles (Table S1). Most proteins (i.e., 411) exhibited either up- or downregulation at a single time point, with only 20 proteins displaying a cyclic regulation profile (strictly up or downregulated over two consecutive light or dark time points). This phenomenon may be attributed to the dynamic fluctuations in proteome phenotype occurring throughout bacterial growth, independent of light conditions (Figure 3).

Interestingly, we observed differential regulation of numerous transcriptional regulatory proteins depending on light conditions, particularly during time points corresponding to dark phases in the LD condition (Table S2). These proteins were involved in various biological processes such as carbohydrate metabolism, oxidative and metal stress response, and virulence factors. While the process of genetic information transfer from DNA to mRNA and then to proteins is well established, there are still significant gaps in our understanding of how modifications at each stage of gene expression can impact downstream cellular activities [29]. This has potential implications for interpreting the impact of the light and dark cycle on *R. rubrum*. While light–dark induced oscillations at the mRNA level are well documented in clock-controlled *Cyanobacteria* such as *Synechococcus* or *Prochlorococcus,* significant discrepancies between diel oscillations at mRNA and protein levels exist [30,31,32]. For instance, in *S. elongatus*, only 14% of its proteome displays LD oscillation [32], while 30% to 60% of the mRNA levels exhibit cyclic profiles [30,33]. 

#### 2.2.2. Impact of Light Conditions on Biological Processes

To decipher the biological interactions and protein network of the differentially regulated proteins, we used the STRING database [34] (Figure 4). Despite achieving satisfactory proteome coverage for *R. rubrum*, we noted that 114 of the regulated proteins were annotated with uncharacterized functions. Interestingly, these proteins were among the most highly regulated across all proteomes, suggesting their potential involvement in various biological processes discussed subsequently. 

This analysis revealed that some of the up and downregulated proteins formed functional networks (Figure 4). These functional groupings were observed during the corresponding dark phases in the LD condition and during the late growth phase. At the beginning of the first dark phase (i.e., 56 h), ribosomal proteins, and proteins involved in oxidative phosphorylation, protein export, and bacterial secretion system were enriched among LL upregulated proteins. At the end of the first dark phase (i.e., 64 h), proteins involved in transcription repair and organic solvent tolerance were enriched among LL upregulated proteins. In the late growth phase (i.e., 96 h), ribosomal proteins and proteins involved in electron transfer activity were enriched among LL downregulated proteins, while proteins involved in miss-match repair system were enriched among LL upregulated proteins. These results suggest that under the LL condition, *R. rubrum* faces higher stress, inducing upregulation of repair systems. On the other hand, under LD conditions, *R. rubrum* might have reduced its metabolic activity (protein synthesis/export and oxidative phosphorylation) during the first dark period then increased it over the late growth phase.

Differential regulation of proteins involved in photosynthesis and oxygenic respiration were observed over time under different light treatments. A total of 34 proteins were found to be up/down regulated in LL compared to LD conditions (Table S3). These proteins were involved in both respiratory and photochemical electron transport systems, photosynthesis reaction centers, bacteriochlorophyll biosynthesis, and ATP synthesis. While no clear distinct metabolic pattern was observed between LL and LD conditions, proteins involved in light-harvesting protein synthesis were upregulated under LD conditions in the early growth phase (i.e., 48 h to 72 h). Under LL conditions, proteins involved in anoxygenic respiration were overall upregulated in early growth and then downregulated in late growth. Since the respiratory and the photochemical electron transport systems of *R. rubrum* are associated [35], further investigation under strict photoautotrophic or heterotrophic growth conditions should better reflect the potential impact of the diel cycle on the energetic metabolism regulation of *R. rubrum*.

Stress-related proteins were also differentially regulated between LL and LD-grown *R. rubrum* cultures (Table S4). The heat shock protein Hsp20, involved in the response to an array of stresses, including hyperthermia, reactive oxygen species (ROS), or heavy metals toxicity [36], was overall upregulated in LD cultures. In contrast, copper/zinc superoxide dismutase was found to be strongly upregulated at 56 h in LL conditions (FC: 4.33, *p*-value = 0.04), correlating with the strong upregulation of NADH ubiquinone oxidoreductase at the same time in LL (FC: 4.85, *p*-value = 0.000095), a major source of ROS [37]. Moreover, we observed LL upregulated transcription repair systems (Figure 4), and a strong upregulation of the SOS-response transcriptional repressor LexA in LL cultures (FC (72 h): 3.89, *p*-value = 0.006, FC (80 h): 35.21, *p*-value = 0.00028). LexA is a DNA repair system repressor, which is cleaved from DNA by RecA when DNA is damaged [38,39]. These results suggest that *R. rubrum* cultures maintained under LL conditions are exposed to higher oxidative stress and light-induced damage when compared to LD cycles [26]. 

The nitrogen regulatory protein P-II was observed at one time point (i.e., 56 h) and was strongly upregulated in the LD condition during the first dark phase (Table S5). This observation refers to the only study presenting rhythmic activity of the uptake hydrogenase, involved in the consumption of H2 produced by nitrogenase hydrogen [23]. Another small subset of proteins associated with virus defense mechanism (e.g., CRISPR system) or host integration factor were differentially regulated between LL and LD conditions (Table S5). In the environment, viral infection was shown to follow a diel pattern, occurring during the night [40]. We also observed four proteins involved in motility (i.e., flagellin) that were upregulated during the dark periods, as well as chemotaxis-related proteins (Table S5). Bacteria utilize motility and chemotaxis (i.e., regulation of motility towards chemical attractants and away from chemical repellents) for optimal growth [41]. Interestingly, diel motility patterns have been observed in the environment [42,43]. Additionally, in the gastrointestinal bacterium *Klebsiella aerogenes*, an endogenously generated, temperature-compensated circadian rhythm in swarming motility has been recently identified [20].

### 2.3. Kai Gene Expression

**Figure 5:**
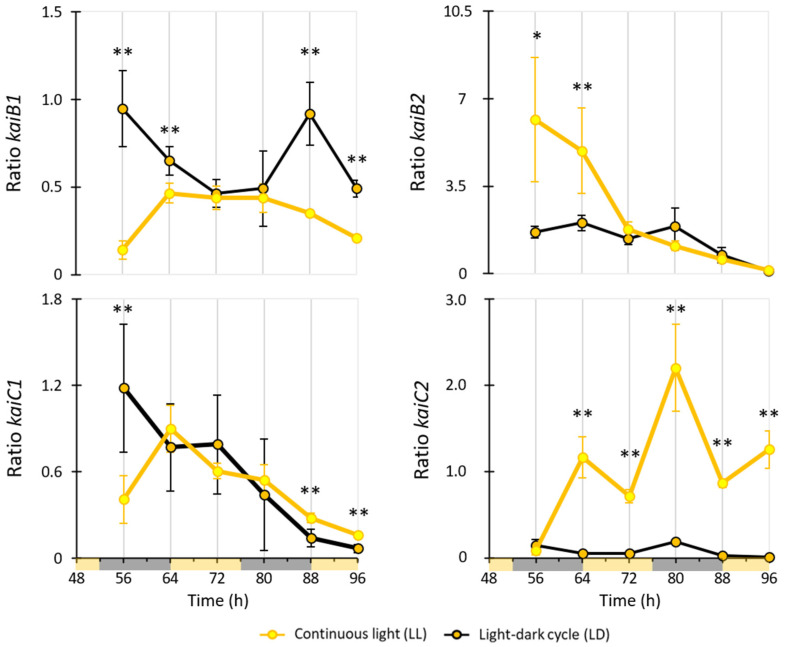
Evolution of the abundance of Kai proteins in *R. rubrum* depending on light conditions. Results were presented as a relative expression ratio of the targeted gene (*kaiB1*, *kaiC1*, *kaiB2*, or *kaiC2*) genes versus a reference gene (*16S rRNA*) for each sample time, in comparison with the expression at T0 (i.e., 48 h). Light and dark phases in the LD condition are represented on the time axis in yellow and grey, respectively. Significant differences between LL and LD samples are shown with a * (*p*-value ≤ 0.1) or ** (*p*-value ≤ 0.05) and were calculated with a *t*-test.

**Figure 6:**
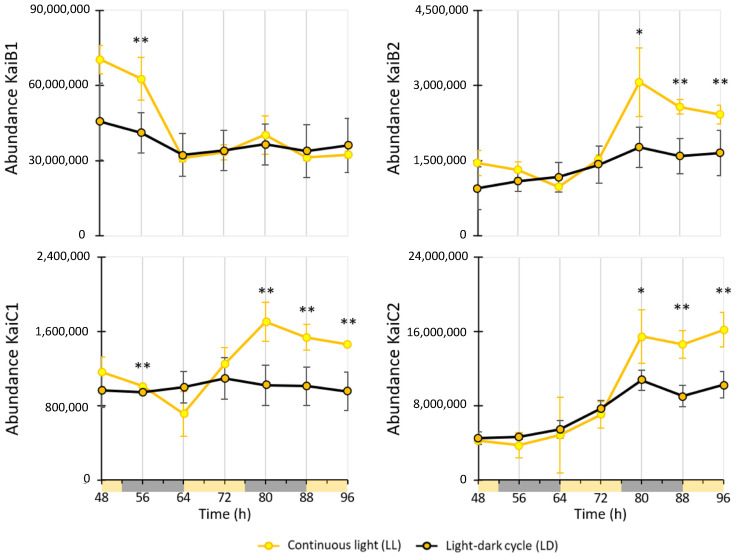
Evolution of the relative abundance of *kai* mRNAs in *R. rubrum* depending on light conditions. Light and dark phases in the LD condition are represented on the time axis in yellow and grey, respectively. Significant differences between LL and LD samples are shown with a * (*p*-value ≤ 0.1) or ** (*p*-value ≤ 0.05) and were calculated with a *t*-test.

Interestingly, the four homologous circadian clock proteins, KaiB1, KaiB2, KaiC1 and KaiC2, were all detected in the SWATH analyses of *R. rubrum*. However, only KaiC1 and KaiC2 were found to be slightly differentially regulated at a single time point (i.e., 88 h) (Table S5). We quantified the expression of the four kai gene homologs using both RT-qPCR and targeted proteomic (Figure 5 and Figure 6). Since measurements were conducted on samples from two distinct experiments (Figure 1), direct comparisons were limited.

At the transcriptional level, the expression of *kai* mRNAs in *R. rubrum* cultures maintained under LL and LD conditions was markedly influenced by the light condition, exhibiting significant fluctuations in relative abundance (Figure 5). In LL conditions, *kaiB2* and *kaiC2* mRNAs were overexpressed and reached maximal abundances three and ten times higher than in LD, respectively. In contrast, *kaiB1* and *kaiC1* were twice more expressed in LD than in LL. 

At the translational level, the expression of Kai proteins under LD conditions showed less variability than under LL conditions (Figure 6). Differences were primarily observed in the late growth phase, where KaiC1, KaiB2, and KaiC2 proteins in LL displayed a higher intensity compared to LD. In contrast, the intensity of LL KaiB1 was higher than LD KaiB1 until 64 h then remained similar. 

While the current data do not allow us to provide definitive conclusions regarding the rhythmic transcriptional or translational regulation of *kai* genes under light and dark periods, they demonstrate that *R. rubrum* actively expresses these genes and that their expression is influenced by light conditions at both mRNA and protein levels. The discrepancy observed between RT-qPCR and MRM-MS results can be attributed to several factors, including post-transcriptional and post-translational modifications, as well as differences in experimental methodologies and inherent limitations of each technique.

Although in *S. elongatus*, *kaiB* and *kaiC* genes exhibit diel rhythmicity at both transcriptional and translational levels [30,44], it is important to note that the primary regulation of the Kai oscillator occurs at the post-translational level through a phosphorylation and dephosphorylation cycle [6]. The role of Kai proteins in *R. rubrum* is still unknown and their function might be related to a timing system that has yet to be identified. Upon closer examination of the position of *kai* genes in the genome of *R*. *rubrum*, we observe that *kaiB2* and *kaiC2* are preceded by the gene Rru_A3293, recently identified as an ortholog of the *kaiA3* gene in *Synechocystis* by Köbler and colleagues [14]. Further investigations into the transcriptional and translational expression of *kai* gene homologs should incorporate *Rru_A3293* to provide a more comprehensive understanding of clock component regulation. Additionally, a more detailed analysis, such as phosphorylated peptide enrichment mass spectrometry, could provide valuable insights into the potential regulation of the Kai oscillator. In addition to phosphorylation, other factors such as protein stability, degradation rates, and translational efficiency could contribute to the observed differences between transcriptomic and proteomic profiles. 

Moreover, it is worth noting that both *kaiBC1* and *kaiBC2* gene clusters are preceded by a histidine kinase containing a PAS domain, namely Rru_A2544 and Rru_A3296, respectively. In *R. sphaeroides,* a similar protein was identified in close proximity to the N-terminal region of the *kaiBC* gene operon. *R. sphaeroides* exhibits a unique pattern of gene expression controlled by oxygen levels (20.5 h under aerobic conditions and 10.6–12.7 h under anaerobic conditions), suggesting a potential role for the histidine kinase protein in sensing oxygen and transducing redox signals to the central clock [22,45]. Thus, it is conceivable that the time-keeping mechanism of *R. rubrum* may be influenced by environmental factors other than light, similar to the role of oxygen in *R. sphaeroides* [22]. Given that purple bacteria coexist within diverse communities, including clock-controlled organisms like *Cyanobacteria*, which rhythmically release organic compounds throughout the day [46], primary production could emerge as another significant driver of rhythmicity in purple bacteria [47]. Future investigations should delve into variations in light and other physicochemical parameters to elucidate the multifaceted role of Kai proteins in *R. rubrum*.

## 3. Materials and Methods

### 3.1. Bacterial Culture Conditions and Sampling

In this study, *R. rubrum* ATCC 11170 was grown in two independent experiences for downstream RT-qPCR and proteomics analyses, respectively (Figure 1). In both experiences, *R. rubrum* was plated on supplemented malate-ammonium medium (SMN) [48] agar Petri dishes at 30 °C in complete darkness. Single colony-forming units were inoculated in 15 mL of SMN liquid medium and pre-cultured at 25 °C, under aerobic conditions and shaken at 150 rpm. For each experience, pre-cultures were grown under two different light conditions: (i) continuous light exposure (LL) and (ii) a 12/12 h light and dark cycle (LD). After 72 h, LL and LD pre-cultures with similar optical density (2.1 ± 0.1 at 680 nm) were selected and 35 µL was inoculated in 150 mL of SMN liquid medium (n = 3). The culture flasks were exposed to similar light, temperature, and shaking conditions as the pre-culture. Every 8 h, 6 mL of bacterial culture were sampled from each flask, from the end of the lag phase (~40 h) until the beginning of the stationary phase (~96 h). Samples were collected at 04:00 p.m. (i.e., 48 h after the start of the experiment), 00:00 a.m. (i.e., 56 h), 08:00 a.m. (i.e., 64 h), 04:00 p.m. (i.e., 72 h), 00:00 a.m. (i.e., 80 h), 08:00 a.m. (i.e., 88 h), and 04:00 p.m. (i.e., 96 h). In total, 7 samples were collected per replicate and per light condition. In the LD condition, light was switched off from 08:00 p.m. to 08:00 a.m. The optical density was measured immediately after sampling by spectrophotometry (680 nm). Cells were harvested by centrifugation (16,000× *g*, 4 °C). Cell pellets were washed twice with BupH^TM^ PBS solution (Thermo Fisher Scientific, Waltham, MA, USA) and stored at −20 °C for downstream RT-qPCR and proteomics analyses.

### 3.2. Protein and RNA Isolation

For the RT-qPCR experiment, total RNA was extracted using the RNeasy Protect Bacteria Mini Kit (50) (Qiagen, Hilden, Germany) following the manufacturer’s instructions. RNA concentration was measured using a Nanodrop spectrophotometer. The Reverse Transcriptase Core Kit (300) (Eurogentec, Seraing, Belgium) was used according to the manufacturer’s instructions to generate complementary DNA (cDNA) from the RNA templates. Briefly, RNA template (15 ng/µL) was mixed with random nonamers (2.5 µM), dNTPs (500 µM of each dNTP), MgCl_2_ (5 mM), EuroScript RT (1.25 U/µL), RNAse inhibitor (0.4 U/µL), RNase free water and reaction buffer. The reverse transcription was performed in a real-time thermocycler with an initial step of 10 min at 25 °C, followed by a reverse transcription step of 30 min at 48 °C and finally, with inactivation of the RT enzyme during 5 min at 95 °C. cDNA was stored at 4 °C until qPCR analyses. 

For the proteomic analyses, proteins were extracted using extraction buffer (guanidinium hydrochloride 6 M; K_2_HPO_4_ 50 mM) and ultrasonication (4 °C, 3 × 10 s, amplitude: 20%, IKA U50 sonicator (Staufen, Germany)). The protein content of the supernatant was assessed using the Bradford assay [49] with bovine gamma globulin as standard. Subsequently, 50 μg of the proteins were reduced with 1.5-dithioerythritol (DTE), alkylated with iodoacetamide (IAA), and precipitated overnight using acetone at −20 °C. The resulting protein pellets were solubilized in 50 mM ammonium bicarbonate containing 1 μg of trypsin and incubated overnight at 37 °C. The trypsin digest was stopped by adding 0.5% formic acid in water (*vol*/*vol* [0.1% final concentration]). Peptides were quantified using the Pierce™ Quantitative Colorimetric Peptide Assay kit (Thermo Fisher Scientific, Waltham, MA, USA). Samples were then stored at −20 °C for subsequent proteomics analyses.

### 3.3. RT-qPCR Analyses

cDNA served as a template for qPCR assay using the Takyon™ Rox SYBR Core Kit blue dTTP (1250) (Eurogentec, Seraing, Belgium). The primers used for qPCR assay were targeting the Rru_A2542 (hereafter *kaiC1*), Rru_A2543 (hereafter *kaiB1*), Rru_A3294 (hereafter *kaiB2*), and Rru_A3295 (hereafter *kaiC2*) reverse transcripts of *R*. *rubrum* (Table 1). The physicochemical properties of each pair of primers were checked using the ThermoFisher Scientific Multiple Primer Analyzer tool [50]. Specificity was confirmed using SnapGene Viewer 4.2.11 (Dotmatics, Bishop’s Stortford, UK), and classic PCR of pure *R. rubrum* culture. Quantification was performed using a StepOnePlus Real-Time PCR System (Thermo Fisher Scientist). The relative concentration of *Kai* was calculated as the ratio of their expression and that of the 16S rRNA using universal primers 518R and 341F (Table 1) according to Pfaffl and colleagues [51] (1). Results were presented as a relative expression ratio of the targeted genes (*kaiB1*, *kaiC1*, *kaiB2*, or *kaiC2*) versus a reference gene (16SrRNA) for each sample time, in comparison with the expression at T0 (i.e., 48 h).

Ratio = (E_target_)^ΔCP target (T0 − Tx)^/(E_ref_)^ΔCP ref (T0 − Tx)^ (1)

where E is the PCR efficiency calculated as defined in Ramakers and colleagues [52] and CP is the crossing point of the amplification curve with the threshold.

qPCR reactions were performed for each DNA sample (n = 3) and targeted gene combination using the SYBR^TM^ Green master mix (Applied Biosystems^TM^, Waltham, MA, USA). Each qPCR consisted in a serial of dilution (2.5, 1.2, 0.6 and 0.3 ng/µL) of cDNA template and qPCR mix following fabricant instruction. After an initial denaturation step at 94 °C during 10 min, 40 cycles of 15 s at 95 °C, 30 s at 60 °C and 30 s at 72 °C were performed followed by a final denaturation step (60 to 90 °C, +0.3 °C/min).

### 3.4. Proteomic Analysis

Protein identification and quantification were performed according to a label-free strategy on a UHPLC HRMS platform (Eksigent 2D Ultra-AB Sciex TripleTOF 6600+). For each sample, the peptides were separated in a 15-cm C_18_ column (YMCC18) using a linear acetonitrile gradient (5 to 35% [*vol*/*vol*] in 75 min) in water containing 0.1% (*vol*/*vol*) formic acid at a flow rate of 5 µL min^−1^. For protein identification, data were acquired in the data-dependent acquisition mode (DDA) for 4 µg peptides on column. Mass spectra (MS) were acquired over the range of 400–1250 *m*/*z* in the high-resolution mode (resolution > 35,000), with a 250 ms accumulation time. MS/MS spectra were acquired over the range of 100–1500 *m*/*z*. The precursor selection parameters were as follows: intensity threshold, 100 cps; maximum precursors per cycle, 90; accumulation time, 25 ms; and exclusion time after two spectra, 15 s. These parameters lead to a duty cycle of 4 s per cycle to ensure that high-quality extracted ion chromatograms (XICs) were obtained for peptide quantification. ProteinPilot Software (v5.0.1—ABSciex, Framingham, MA, USA) was used to perform database searches against the UniProt database, restricted to *R. rubrum* ATCC 11170 entries. The search parameters included differential amino acid mass shifts for carbamidomethyl cysteine, all biological modifications, and missed trypsin cleavage sites. 

For whole-protein relative quantitation analyses, the instrument was set to the SWATH mode. Briefly, 100 incremental steps were defined as windows of variable *m*/*z* values over a 400–1250 *m*/*z* mass range. The MS/MS working time for each window was 50 ms, leading to a duty cycle of 5 s per cycle. The ion chromatogram of the top six fragments of each peptide was extracted, and their area under the curve was integrated. PeakView^®^ software (version 2.1.0.11041, ABSciex, Framingham, MA, USA) was used for the SWATH processing of all proteins identified considering an FDR below 1% (as determined by ProteinPilot). The retention time (RT) was recalibrated automatically with PepCalMix standard (ABSciex, Framingham, MA, USA), with retention times in the range of 20–85 min. The intensity of peptides was individually normalized based on a summed area of all peptides for each sample. Only proteins quantified with a minimum of 2 peptides were considered. A non-supervised Pareto-PCA analysis was performed according to the area of protein curve to discriminate comparative groups (MarkerViewTM MarkerView™ 1.2.1 ABSciex, Framingham, MA, USA). For proteome comparisons, only fold changes higher than 1.5 (upregulated in LL compared to LD) or lower than 0.66 (downregulated in LL compared to LD) and having a *p*-value lower than 0.05 were further considered. The function of each differentially abundant protein was checked manually by using Uniprot, and the NCBI database.

The MRM analyses were performed using a QTRAP 6500+ instrument (SCIEX) fitted with an electrospray ionization source (150 °C, 4500 V). Transition selection and MRM method optimization were performed using the Skyline software (20.2.0.343 MacCoss Lab, Seattle, WA, USA) on protein extracted from *R. rubrum* grown under light and dark cycle. The best transitions (y or b ions) were chosen for each peptide and at least two peptides were analyzed for each protein. Peptides were separated on a C18-reversed-phase column (YMC TriArt C18, 0.3 mm, 150 mm) and eluted using a gradient of 5–35% acetonitrile with 0.1% formic acid over 20 min at a flow rate of 5 µL/min. MRM data were acquired in the scheduled mode with a two-minute retention time window and a maximum cycle time of 1.5 s. Skyline software (20.2.0.343 MacCoss Lab) was used for visual inspection of MRM data and area under the curve integration. Peak picking for each peptide was manually refined using the transition intensity ratio and retention time as leading parameters. The intensity of all transitions was summed up for each peptide. Protein abundance was obtained as the average of the Ln-transformed area under the curve of each target peptide detected in the three samples.

## 4. Conclusions

Deciphering the influence of the diel cycle on purple bacteria holds significant promise for enhancing our understanding of their ecological roles, optimizing their application in biotechnology, and unraveling the origins and evolution of prokaryotic clock mechanisms. This study represents a pioneering effort in providing a comprehensive molecular analysis of the diel cycle’s impact on the photoheterotroph *R. rubrum*, a species previously identified as a potential candidate for a role in the circadian rhythm due to the presence of *kai* genes. Our findings unveil a noteworthy influence of light conditions on protein expression, particularly during the early growth stage.

Numerous proteins involved in transcriptional regulation, energy conversion, stress response, and, to a lesser extent, motility, viral defense and chemotaxis exhibited differential regulation under continuous light exposure compared to a light and dark cycle. Light emerged as a key factor shaping the expression of kai genes at both the transcriptional and translational levels, hinting at their potential involvement in a time-keeping mechanism that remains to be fully elucidated. 

Moving forward, there are several promising avenues for further research to enhance our understanding of *R. rubrum*’s response to the diel cycle. One pivotal area of investigation involves capturing the diel transcriptional cycle of *R. rubrum* under controlled conditions, particularly through continuous culture in a chemostat. In addition to transcriptomic studies, integrating phosphoproteomics into the workflow holds substantial potential. This advanced technique can reveal the phosphorylation events within the proteome, providing a more nuanced view of the regulatory dynamics associated with the Kai oscillator. Such investigations may uncover key signaling pathways and refine our understanding of temporal adaptations at the post-translational level.

Another promising avenue is the exploration of upstream and downstream genes associated with the Kai oscillator to unravel the broader regulatory network governing circadian rhythms in *R. rubrum* and potentially in other bacterial species. This comprehensive approach has the potential to unveil novel regulatory mechanisms and broaden our understanding of the multifaceted roles of circadian rhythms in bacterial physiology.

## Figures and Tables

**Table 1:** Primer sequences targeting *kai* gene homologs in *R. rubrum*.

	Primers	
	Forward (5′–3′)	Reverse (5′–3′)
16S rRNA	CCTACGGGAGGCAGCAG	ATTACCGCGGCTGCTGG
kaiB1	GCCCACGGAAACTAACGCTC	GTTCCGCGCAAATCCGTTC
kaiB2	GATGTGATCGACAGTCCCGC	AGATCAAGGATGCGGCACAC
kaiC1	TTCAGCGTTCTTCCCGTCTC	GACCAGGATGCTTGATCCCC
kaiC2	TCTTTTCCGCCCAGTTCCTG	TCGACGAAGCTCCATTTCCC

## Data Availability

Data is contained within the article and Supplementary Material.

## Supplementary Materials

The following supporting information can be downloaded at: https://www.mdpi.com/article/10.3390/ijms25052934/s1.
