# Supplementary material for: Diel Cycle Proteomics: Illuminating Molecular Dynamics in Purple Bacteria for Optimized Biotechnological Applications
Source: Int J Mol Sci. 2024 Mar 2;25(5):2934. doi: 10.3390/ijms25052934 (PMC10931921; doi:10.3390/ijms25052934)
Supplement: Supplementary file 1 [file ijms-25-02934-s001.zip › ijms-2878906-supplementary.pdf]

Table S1. Up- and downregulated proteins in *R. rubrum* classified into protein regulation profiles.
Up arrow and down arrow correspond to upregulation or downregulation, respectively, in LL compared to LD at each sampling time.

	Regulation profile	48h	56h	64h	72h	80h	88h	96h	Protein number
Upregulated	Single	↗	-	-	-	-	-	-	11
	Single	-	-	-	↗	-	-	-	8
	Single	-	-	-	-	-	-	↗	11
	Consecutive	↗	-	-	↗	-	-	-	1
	Single	↘	-	-	-	-	-	-	10
	Single	-	-	-	↘	-	-	-	19
	Single	-	-	-	-	-	-	↘	26
	Consecutive				↘			↘	2
Downregulated	Single	-	↗	-	-	-	-	-	74
	Single	-	-	-	-	↗	-	-	13
	Consecutive	-	↗	-	-	↗	-	-	1
	Single	-	-	↗	-	-	-	-	13
	Single	-	-	-	-	-	↗	-	19
	Consecutive	-	↗	↗	-	↗	↗	-	2
	Single	-	↘	-	-	-	-	-	107
	Single	-	-	-	-	↘	-	-	42
	Single	-	-	↘	-	-	-	-	10
	Single	-	-	-	-	-	↘	-	48
	Consecutive	-	↘	-	-	↘	-	-	12
	Consecutive	-	-	↘	-	-	↘	-	2

Table S2. Transcriptional regulatory proteins regulated depending on light conditions in *R. rubrum.*

Transcriptional regulatory proteins

48h

Regulated proteins	FC
Transcriptional regulator, DeoR family	2.13
Transcriptional regulator, XRE family	1.74
Heat-inducible transcription repressor hrcA	0.57

56h

Regulated proteins	FC
Transcriptional regulatory protein	2.21
Transcriptional regulator, MucR family	1.55
Periplasmic binding protein/LacI transcriptional regulator	0.65
Two component transcriptional regulator, winged helix family	0.64
Transcriptional regulator, MarR family	0.62
Probable transcriptional regulatory protein Rru_A1086	0.57
Transcription antitermination protein nusG	0.56
Periplasmic binding protein/LacI transcriptional regulator	0.50
Transcriptional regulator, GntR family	0.29

64h

Regulated proteins	FC
Transcriptional regulatory protein	1.94
Transcriptional regulator, TetR family	1.85
Transcription-repair coupling factor	1.69
Transcriptional regulator, ArsR family	0.38

72h

Regulated proteins	FC
None	

80h

Regulated proteins	FC
Transcriptional regulatory protein	3.80
Periplasmic binding protein/LacI transcriptional regulator	0.61
Periplasmic binding protein/LacI transcriptional regulator	0.60
Periplasmic binding protein/LacI transcriptional regulator	0.56

88h

Regulated proteins	FC
Transcriptional regulatory protein	3.16
Two component transcriptional regulator, winged helix family	1.77
Transcriptional regulator, BadM/Rrf2 family	0.63
Transcriptional regulator BolA	0.63
Transcriptional regulator, XRE family	0.56
Transcriptional regulator, XRE family	0.42
Two component transcriptional regulator, winged helix family	0.30

96h

Regulated proteins	FC
None	

Table S3. Proteins involved in energy conversion regulated depending on light conditions in *R. rubrum*.

Proteins involved in energy metabolism

48h

Regulated proteins	FC
Cytochrome c, class II	1.69
Ubiquinol-cytochrome C chaperone	1.62

56h

Regulated proteins	FC
NADH-ubiquinone/plastoquinone oxidoreductase, chain 6	4.85
4Fe-4S ferredoxin, iron-sulfur binding	3.50
ATP synthase epsilon chain	1.95
ATP synthase subunit alpha	1.92
Ubiquinone biosynthesis protein COQ7	1.80
NADH-quinone oxidoreductase subunit B	1.67
NADH-quinone oxidoreductase subunit I	1.57
NADH:flavin oxidoreductase/NADH oxidase	1.57
ATP synthase gamma chain	1.50
PfkB	0.64
NADH:flavin oxidoreductase/NADH oxidase	0.61
NADH-plastoquinone oxidoreductase, chain 5	0.59
Cytochrome c, class II	0.59
Ferredoxin--NADP+ reductase	0.58
Chlorophyllide reductase iron protein subunit X	0.57
PfkB	0.54

64h

Regulated proteins	FC
Photosynthetic reaction center, H-chain	2.84
Protoporphyrin IX magnesium-chelatase	0.48
4Fe-4S ferredoxin, iron-sulfur binding	0.17

72h

Regulated proteins	FC
ATP synthase subunit c	9.99
4Fe-4S ferredoxin, iron-sulfur binding	2.03
Chlorophyll synthase	0.63
Photosynthetic reaction center, H-chain	0.37

80h

Regulated proteins	FC
4Fe-4S ferredoxin, iron-sulfur binding	4.63
Cytochrome B561	2.12
Chlorophyllide reductase subunit Z	1.69
Photosynthetic complex assembly protein	1.51
Pyruvate ferredoxin/flavodoxin oxidoreductase	0.61
Ferredoxin	0.59
Cytochrome-c peroxidase	0.57
Cytochrome c2	0.46
Cytochrome c, class II	0.45
Cytochrome c'	0.39

88h

Regulated proteins	FC
Cytochrome C oxidase, mono-heme subunit/FixO	0.66
NADH-quinone oxidoreductase subunit N	0.61
Ferredoxin	0.61
Ubiquinol-cytochrome c reductase iron-sulfur subunit	0.59
Cytochrome-c peroxidase	0.58
Phenylpyruvate decarboxylase	0.57
Chlorophyll synthase	0.56
4Fe-4S ferredoxin, iron-sulfur binding	0.56
Cytochrome c oxidase cbb3-type, subunit I	0.54
PfkB	0.53
2-desacetyl-2-hydroxyethyl bacteriochlorophyllide	0.52
Photosynthetic complex assembly protein	0.51
Cytochrome B561	0.32
4Fe-4S ferredoxin, iron-sulfur binding	0.05

96h

Regulated proteins	FC
Cytochrome C oxidase, mono-heme subunit/FixO	0.66
4Fe-4S ferredoxin, iron-sulfur binding	0.66
Cytochrome c'	0.59
Cytochrome c2	0.58
Cytochrome c, class II	0.58
Cytochrome-c peroxidase	0.55
ATP12 ATPase	0.45
Photosynthetic reaction centre M subunit	0.24
4Fe-4S ferredoxin, iron-sulfur binding	0.10

Table S4. Stress response proteins regulated depending on light conditions in *R. rubrum*.

Stress response proteins

48h	
**Regulated proteins**	**FC**
Transcriptional regulator, XRE family	1.74
Heat-inducible transcription repressor hrcA	0.57
Heat shock protein Hsp20	0.34

56h	
**Regulated proteins**	**FC**
Copper/Zinc superoxide dismutase	4.34
Heat shock protein Hsp20	2.12
Succinate semialdehyde dehydrogenase	1.96
Rubrerythrin	0.66
Glutathione S-transferase family protein	0.63
Stress protein	0.50
Superoxide dismutase	0.35

64h	
**Regulated proteins**	**FC**
Organic solvent tolerance proteintA-like	4.04
Transcriptional regulator, TetR family	1.85

72h	
**Regulated proteins**	**FC**
LexA repressor	3.89
Heat shock protein Hsp20	0.53

80h	
**Regulated proteins**	**FC**
LexA repressor	35.21
Glutathione S-transferase-like	0.60

88h	
**Regulated proteins**	**FC**
Transcriptional regulator, XRE family	0.56
Heat shock protein Hsp20	0.52
Transcriptional regulator, XRE family	0.42
Glutathione synthetase	0.38

96h	
Glutathione S-transferase-like	14.01
DNA mismatch repair protein MutL	2.24
Rubrerythrin	0.65
Aldehyde dehydrogenase	0.65
Heat shock protein Hsp20	0.53

Table S5. Proteins involved in viral injection/defense, motility, chemotaxis, nitrogen cycle, and circadian clock regulated depending on light conditions in *R. rubrum*.

Miscellanous

48h

Regulated proteins	FC
CRISPR-associated protein, Cse4 family	1.51

56h

Regulated proteins	FC
Methyl-accepting chemotaxis sensory transducer	2.48
Chemotaxis sensory transducer	1.53
Flagellin-like	0.66
Integration host factor subunit alpha	0.63
Phage shock protein A, PspA	0.60
CRISPR-associated protein, CT1974	0.59
Flagellar basal-body rod protein FlgC	0.52
Nitrogen regulatory protein P-II	0.18

64h

Regulated proteins	FC
Flagellin-like	0.63

72h

Regulated proteins	FC
CRISPR-associated protein, Cse4 family	1.57

80h

Regulated proteins	FC

88h

Regulated proteins	FC
Flagellin-like	0.30
Circadian clock protein KaiC	1.5
Circadian clock protein KaiC	0.6

96h

Regulated proteins	FC
Methyl-accepting chemotaxis sensory transducer	2.18
Chemotaxis response regulator protein-glutamate methylesterase 1	0.62
